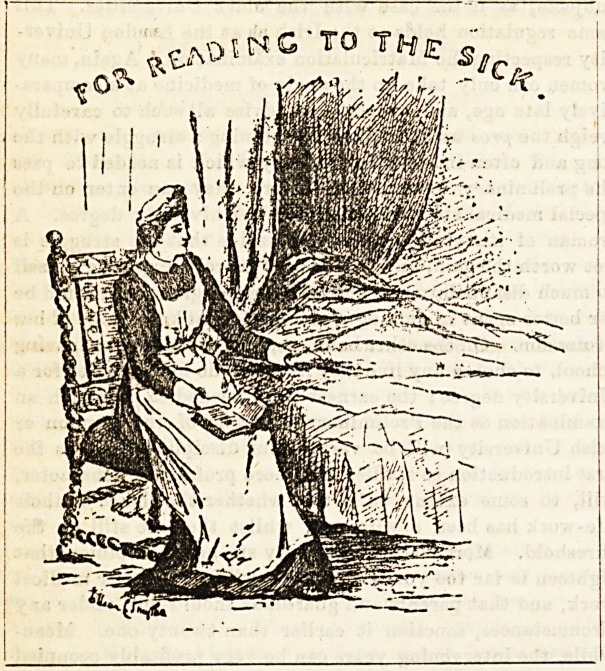# The Hospital Nursing Supplement

**Published:** 1892-09-10

**Authors:** 


### "The Hospital" Nursing Mirror.

Being the Extra Nursing Supplement of "The Hospital" Newspaper.

Contributions for this Supplement should be addressed to the Editor, The Hospital, 140, Strand, London, W.C., and should have the word "Nursing" plainly written in left-hand top corner of the envelope.

### En Passant.

MIDWIVES REGISTRATION.—The Select Committee has published its report, and from the evidence taken some legislative provision is declared desirable. A continuation of the inquiry is recommended during the next Session of Parliament.

CRUMPSALL INFIRMARY, MANCHESTER.—At the recent examination of probationary nurses, after the summer course of lectures, thirteen probationers received first class certificates. In this training school for nurses over forty pupils are constantly under instruction. Miss Hanan, the Lady Superintendent, whose health compelled some months' absence, has now returned to the infirmary.

NURSES' CO-OPERATION.—This Association continues to grow and to prosper, as it deserves. Although this is generally considered "the flat season" by private nurses, and a certain proportion of them wisely select it as the best time to take their well-earned holidays, yet those who have remained in town have had unusually numerous demands for their services this summer. There will be a few vacancies in November, but only a few; and we should advise thoroughly-trained nurses wishing to join to make their applications during the first week in October.

LONG LIFE FOR NURSES.—It is rather a pet saying nowadays, "Oh! *I* shall never live to be old. I am sure I need not trouble about making provision for the days which I am not likely to see." But this view is certainly not in accordance with the conclusions arrived at by Dr. Arlidge, who says in his recently-published book, the mortality among "the unoccupied classes" is particularly heavy. Therefore, the deduction is clear that the "occupied classes" have the best chance of long lives, and to make them also healthy and profitable ones, assuredly lies in the hands of each individual worker.

LADY STUDENTS.—Now that Aberdeen has opened its schools to women, and Edinburgh is evidently inclining towards an equally liberal] policy, we feel that for girls, medical education is being made daily more smooth. But when they get into the wards as dressers or clerks to a young resident medical officer, there are still little difficulties to overcome. The wise and experienced visiting physician, who at first patiently "puts up with" the lady students, is often found taking an increasing interest in the intelligent following which accepts his teaching so eagerly. But the young man, accustomed hitherto to fellow students of his own sex, is much perplexed at finding himself attended by half-a dozen young women. He either, with characteristic awkwardness, adopts a brusque tone and a semi-defiant manner, or he lets his instinctive chivalry have its way and treats his dressers as ladies, but feels uncomfortably incapable of reprimands or criticisms. Time will doubtless evolve for these young medical officers a code of etiquette which will decide their little difficulties for them. It will settle a comical detail, which we heard one of them speak of some time ago, he said, "All the time I was holding that post I tried to solve an enigma, which puzzled me from my first day in the wards until the hour when I regretfully left them. Ought I, as an official, to walk through the doors first, followed by my pupils, or should I, as an English gentleman, establish a precedent and open the door for them, as I naturally should for any other women?"

THE ADELAIDE HOSPITAL, DUBLIN.—We have received the annual report of this hospital, and are glad to read of its prosperity. The sum of £1,062 6s. 5d. was contributed by the Hospital Sunday Fund Committee, which expressed great satisfaction with the general management and existing nursing arrangements. Although no patients are admitted for treatment in the wards, unless they profess "the principles of the Protestant Reformed Churches," the out-patient dispensaries "are open daily for the relief of the sick of all denominations," and an average daily attendance of 100 poor persons is recorded.

WOMEN AND FIRE DRILL.—In some hospitals and infirmaries it is the wise custom to teach the nurses how they should act in case an alarm of fire should be given. Let us hope the lessons will have practical value, if so sad a disaster ever occurs; and our English girls will then prove themselves as capable of prompt action as did the sisters at the Refuge at Anglet the other day. A fire broke out near to them, and 200 women immediately appeared on the scene, and by their able management of swiftly supplied pails of water, they actually subdued the flames and averted a great catastrophe.

TALL OR SHORT?—There is often some question asked on this subject by would-be nurses, especially those young ladies whose excessive stature, or else the lack of it, renders them personally interested in the subject. Many Matrons prefer their nurses to be of medium height, finding that their best workers are generally of moderate size. A tall Matron or Sister has certainly an imposing and graceful appearance, but for every-day ward routine, the shorter, broader-built woman, is undoubtedly the best. A very small personage has much to contend with, but if she is well proportioned and of sound health, she frequently proves as fit for her profession as the lady who stands 5 feet 6 inches in her stockings.

A SEASONABLE SUGGESTION.—How many little patients attending the hospital every week are at last discharged with "You need not bring him up again, Mrs. Brown; I can't do anything more, all he wants now is a thorough change. Get him away to the country or to the sea if you can, he'll be all right then in a month or two." If the child be a cripple, or feeble, or helpless, this is a difficult task; he is ineligible for any "Country Holiday Fund," and therefore provision for him must be made through some other source. The change which people "who fare sumptuously every day" find most desirable for their own offspring, from a health point of view, is absolutely necessary for the sickly child, whose days are passed in bad air and amidst insanitary surroundings. Therefore, when our wealthier brethren return from their successful seaside holidays, let them send pitying thoughts towards the invalids in the courts and alleys of the town. If they have neither the time nor the knowledge for making inquiries; not only for cases needing help, but for the best places in which to deposit these poor mites, for the desirable "thorough change," they can send a cheque to the Invalid Children's Aid Association, 18, Buckingham Street, Strand, and the committee will take all the trouble and responsibility off their hands. There is no fashionable month for these babies, they will derive quite as much benefit from visits paid in September and October, as in July or August! The I.C.A.A. has already 2,020 names on its books, but we believe it is always willing to attend to the claims of any new applicants.

### Lectures for Asylum Attendants.

By William Harding, M.B.

### I.—INTRODUCTORY (*Concluded*).

Those cells or communicating fibres which are employed in the production of the intellectual faculties may be diseased. The result is that these faculties become impaired or destroyed, and so delicate are the structures affected that a very slight change in them makes a serious alteration in the functions they perform. We have said that an idiot is an individual with an imperfectly developed brain; an imbecile is an individual whose brain is more highly developed than that of an idiot, but yet does not reach the ordinary standard. The possessor of a normally developed brain in healthy working order is said to be sane, or in other words to have a sound mind. If those parts of the brain concerned in the production of the intellectual faculties be diseased then we say that the possessor of such a brain is insane, or that he has not a sound mind. It is with persons so afflicted that we have to deal.

Any of the intellectual faculties may be impaired. We will find patients in whom reason, will, memory, self-control, emotions are affected, and in whom the animal instincts are altered or perverted. These are merely symptoms which show us that the highest functions of our brains are disordered. The lunatic cannot be held responsible for many of his doings; they are the result of disease. From his point of view, behaviour which to us appears utterly wrong, is to him perfectly correct and justifiable. His will-power or self-control may be impaired, and hence he performs purposeless acts, or gives way to violent or destructive impulses. Thus we account for delusions; for irrational language and conduct; for incoherent speech and noisy excitement; for depression and suicidal tendencies; for dogged obstinacy and facile changeableness; for the forgetfulness which makes the past of but a minute ago an utter blank. Hence, also, we have the altered appetites, the dirty habits, and the indecent practices to which some lunatics are subject. They are all indications of the mischief at work in the higher brain centres.

A delusion is a "false belief arising from diseased mental action," as when a pauper believes that she is the Queen of England. An hallucination is a false impression received through one of the special senses, and if the individual believes in the reality of the impression, then the hallucination becomes a delusion. Thus a person may see animals in a room where none exist, but by the exercise of his reason he may convince himself that the object which he thinks he sees is, in reality, non-existent. If, however, he believed that the animals are actually present in the room, then the hallucination has become a delusion.

Lunatics may be divided into four great classes. An insane person may be completely changed from his state when in health as regards spirits, manner, and behaviour generally. From being quiet and retiring he may become the very reverse. He may be restless, excitable, and talkative; perhaps incoherent, violent, and destructive. There is exaggerated action of some of his mental powers, an impairment of others, and a great want of self-control. His whole condition is that of exaltation or undue action. States of exaltation form the first of these great classes. The individual may have delusions, but they are not the outstanding feature of his illness.

In contrast to this condition, however, the patient may be dull, depressed, and utterly miserable. There is to him no pleasure in life. It is acute pain merely to exist, and he may seek to put an end to it all by starvation or otherwise. Often there are most distressing delusions, though these are not essential. He feels that some awful doom is impending. The unpardonable sin has been committed, and the wretched sufferer ever sees hell yawning open before him, while ghostly denunciations ring constantly in his ears. He spends his days and most of his nights in a fearful waking nightmare. It is no wonder that suicidal impulses are frequent. Such cases as these form typical examples of the "states of depression."

Many cases lie between the extremes of exaltation or depression, but may be referred to one or the other as their symptoms indicate.

A third great class is formed by those who have delusions, but who show no marked alteration in their conduct either towards exaltation or depression. This class might be called "Delusional Insanity." The first and third classes together may be said to be suffering from "mania," the second from "melancholia."

The fourth great class consists of "states of mental weakness" occurring in persons whose minds at one time were up to the average standard, but, as the result of injury or disease, have become feeble. This class comprehends the majority of the chronic residents in our asylums. Their mental condition varies from slight enfeeblement to the living death of the utterly demented, who live the life of vegetables, and require to be cared for like babes.

The mental condition of the epileptic and general paralytic can, at the various stages in their careers, be referred to one or other of these classes; but, as they have special characteristics of their own, they will be dealt with separately when we speak of the management of patients.

We have all experienced how the mind is affected by the general health of the body, and we know that the state of the mind reacts upon the system. An unhealthy body is a great strain upon a sound mind. We know how differently things appear to us when we are in good health, compared with what they do when we are ill and irritable. A man suffering from the gout is not situated in the best possible circumstances for taking a calm, patient, and reasonable view of his surroundings. Loaded intestines and an overworked stomach are the cause of much misery and mental pain in this world. Cold and uncleanliness are not conducive to comfort, and when we are uncomfortable our mental peculiarities do not show up in a favourable light. We have all felt that the state of the atmosphere had an influence upon our feelings, and that our views of men and things were much influenced by our surroundings. All these things influence us, and in a greater or less degree also exert their power upon the insane. We have our reasoning powers, and ought to know how best to maintain the conditions necessary for our health; the insane in many cases are quite unable to do this, and yet it is most essential that they should be placed under the best hygienic conditions possible. They must be thought for and provided for. The nurse must remember that in many cases she must think and act for the patient. She should be acquainted with the state of each individual under her care, as regards her clothing, appetite, excretions, and cleanliness, and she should see that the ventilation and warmth of her ward are kept up to the standard. The subjects we will first consider will be—1. Ventilation. 2. Warmth. 3. Cleanliness. 4. Food. 5. Excretions.

### Where to Go.

The Congress and Health Exhibition of the Sanitary Institute will be held from September 12th to October 8th, and special railway arrangements will enable visitors to go to Portsmouth and back for single fares. The Conference of Ladies takes place on the second day, Tuesday, 13th inst when papers relative to Domestic Hygiene, Sanitation and Food, with Special Relation to the Sick, will be read.

### How to Become a Medical Woman.

THE days when there was only one school set apart for teaching women the science of medicine are now passed, and the would-be woman-doctor has carefully to weigh in the balance the various advantages and disadvantages of the schools in London, Edinburgh, and Glasgow. It is with a view to setting before the would-be student, in as clear and concise a form as possible, the information she needs to help her to make her choice, and which she would otherwise have to laboriously collect for herself from the prospectuses issued by the schools, that we proceed to describe how to become a woman-doctor. Our aim is merely to set the bare facts forth, and to leave the decision to our readers. We have, personally, no bias whatsover towards one school rather than another.

The first point the student must decide is the amount that she can afford to spend on the course of study required to obtain a qualification to practice. Until lately, five years had to be devoted to obtaining a University diploma, and only four years for the qualification granted by the Society of Apothecaries and by the Scotch and Irish Colleges. The Medical Council, however, has now decided that in every case five years must be devoted to medical study, and this new arrangement affects all who were not registered as medical students before January, 1892. This extra year, involving as it does, classes in fresh subjects, will increase somewhat the total cost of such qualification as those of the Apothecaries' and Scotch and Irish Colleges, but will not affect the University curriculum, which has covered five years all along, and hence remains unchanged. In addition to the cost of the lectures, &c., which form the medical course, the amount required in fees for examinations only varies very much. The cost of the London qualification; that is, the M.B. degree, is higher than the Irish, though the actual expense of the latter is greater, except for students in Dublin, as it is necessary to go over to Ireland for each examination. The following table will show at a glance the fees charged by the different examining bodies for the triple qualification:—

| | | |
|---|---|---|
| Degree of M.B. and B.S. of London University ...... | £20 | 0 |
| Degree of M.B. of Irish University...................... | 6 | 0 |
| Diploma of College of Physicians and Surgeons, Ireland........................................................ | 42 | 0 |
| Diploma of College of Physicians and Surgeons, Edinburgh .................................................. | 26 | 0 |
| Diploma of Faculty of Physicians and Surgeons, Glasgow ..................................................... | 26 | 0 |
| Diploma of Society of Apothecaries, London............ | 10 | 10 |

It must be added that many who, in the first instance, take only one of the above diplomas, add, later on, an M.B. or M.D. degree, acquired at Brussels or elsewhere abroad.

In coming to a decision respecting the qualification sought, the student should take into account her previous education and the age at which she can begin her studies, as well as the actual cost in money. Thus, anyone who has been educated at a high school or college, or who has passed, say, the London Matriculation examination, will be much better able to work without undue strain for a University degree than one who has left school some years, and has been occupied chiefly with domestic duties in the meanwhile. For such a one to have to go back to school studies and to work for a stiff preliminary examination like the Matriculation of the London University, which comprises a great variety of subjects, all of which must be passed at once, or the candidate will be "plucked," would be a mistake. We may justly hope, however, that this defect will presently be remedied when the scheme for the new Teaching University in London is matured, and that it may be possible to pass a less exacting entrance examination, and a more severe one later on, when the student is free to specialise in certain subjects, as is the case with the older Universities. This same regulation holds at the Irish as at the London University respecting the Matriculation examination. Again, many women can only take up the study of medicine at a comparatively late age, and we strongly advise all such to carefully weigh the *pros* and *cons* before beginning a struggle with the long and often intolerable drudgery which is needed to pass the preliminary examination before they can enter on the special medical studies needed for a University degree. A woman of mature age may well decide that the struggle is not worth the gain, and that she is merely exposing herself to much disappointment, and wasting energies which could be far better spent in gaining a more practical knowledge of her profession. On the other hand, those who are free, on leaving school, to choose any line they like, will do well to enter for a University degree; the earnest work needed to pass such an examination as the Preliminary Scientific of the London or Irish University will be very useful discipline, and, as the first introduction to studies of a more professional character, will, to some extent, be a test whether the choice of their life-work has been a wise one, whilst they are still on the threshold. Moreover, we are very strongly of opinion that eighteen is far too young for any girl to begin her medical work, and that parents and guardians should not, under any circumstances, sanction it earlier than twenty-one. Meanwhile, the intervening years can be very profitably occupied in studying sciences, such as chemistry—a thorough knowledge of which, both organic and inorganic, is necessary, as also magnetism and electricity, &c.; nor would three years be at all too much to devote to these subjects, if anything like a thorough knowledge of them is to be obtained. It is indeed strange that any parents or guardians can be found who cheerfully allow their young daughters to take up medicine at eighteen. It does not require a personal experience of the dissecting-room, but merely the exercise of a very little imagination, to realise how very trying is the work that has to be carried on there, and for many consecutive hours, and how harmful such influence may well prove to a young girl. It seems, however, that this slight effort of imagination is more than some parents can make. The present writer would urge strongly on all women who can to aim at a University degree as being the best and highest qualification it is possible to obtain, and therefore a worthy goal for their ambition, and the very best possible preparation for the responsible work they look forward to perform in the future. There is only one drawback to such a qualification, and that is one of women's own making, and which it rests with her to remove. The minds of many women do not seem able to expand in all directions, but the training of some faculties causes the warping of others. Women have proved themselves thoroughly capable of acquiring the highest scientific knowledge, but there this power seems to cease, and she who is able to apply her science practically has not yet made her appearance. With medical women, in particular, this tendency to allow science to prevail over practice should be most carefully guarded against. After all, the highest aim of a medical man's or woman's skill, is to cure those so-called "slight ailments" to which almost every one in the present age is only too liable. It has been justly said, though it may sound paradoxical, that "anyone can perform an out-of-the-way operation or cure an uncommon disease," but what we want are those who can cure the indefinable but very real troubles which often make daily life a well-nigh unbearable burden, and the necessary work and daily bread-earning an almost insuperable difficulty. We would fain see "women medicals" to the fore in such work, especially as their own sex are the chief sufferers. If the elaborate study of science which is needed to attain a University degree exhausts the energies of their minds, and ends by merely making them scientific and nothing more by preventing the application of their science to every-day life, then it were better not to have this cumbersome science, and to replace it with practical common-sense. It rests with women themselves to see that a University course has not this warping, narrowing effect on their minds, but is used to equip them the better for their work.

*(To be continued.)*

### UNBELIEF.

In thinking of, or addressing sick persons, one is apt to picture them to ourselves as believers in God, who only require to be taught a little to realise that the Almighty sends trials and afflictions for our good, and that He will help us bear them with patience. Experience shows us, unfortunately, that this is not always the case, there are some unhappy ones who scoff at religion and declare there is no God, no devil, no evil, and no good outside themselves; nothing is real but the ideas of their own brains. We can but pity such miserable beings, who crucify their own souls and stifle back the deepest craving of the human heart. There is an immortal part in every one of us which needs someone to share its pain, and which utters an irrepressible cry for sympathy in sorrow, especially in our last agony. "O, my friends, the approach of death is very dreadful," said a great man when lying helpless from paralysis, but he was a good one also, and immediately added, "Let us learn to derive our hope only from God, and in the meantime be kind to one another." The deathless part of us is ever seeking the comfort of hope by which to live, and strength to endure the daily pain which is our portion, and yet men will not go to the source from which to obtain these blessings, and obstinately turn aside and hew out for themselves cisterns, broken cisterns, which can never hold the water of life for them. There are many who know the truth, but do not love it enough to gain comfort, while other hapless beings have scarcely heard of Christ who bought them. To one and all of these sufferers we say, Bear with us, dear friends, while we point out to you a more excellent way. Holy Scripture tells us that it is the foolish body who hath said in his heart there is no God. Let us not, then, swell the ranks of these madmen, but claim our birthright as the sons of Him who sent our Elder Brother into the world to be our pattern, by which we may live, by which we may dare to die. With all our striving we can reach no higher than the Son of God, and if we have sense enough to seize on Him, to cling tightly to His pierced hands, or with humility kiss the hem of His garment, we shall be whole. Body and soul alike can gain their health from this Great Physician. His own sufferings have made Him tender and compassionate to the sick and feeble, and we may carry our troubles to Him. He will soothe our agonies, He will calm the tumult in our breasts if we will only believe all things are possible to him that believeth. Let our prayer be ever, "Lord, I believe. Help Thou mine unbelief."

### Everybody's Opinion.

*[Correspondence on all subjects is invited, but we cannot in any way be responsible for the opinions expressed by our correspondents. No communications can be entertained if the name and address of the correspondent is not given, or unless one side of the paper only be written on.]*

### METROPOLITAN FEVER HOSPITALS.

We have just received a letter from a trained and certificated nurse who has lately been associated with the Eastern and Northern Fever Hospitals, and we are glad to publish her remarks in connection with the subject of visitors to patients, referred to by us in last week's "Nursing Mirror." "Sir, you were justly informed with regard to enquiries about patients. They are answered at once, and most fully. No only are parents given every information about their children, but they are always permitted to see them when they are particularly anxious about their being kept in a long time." The writer adds that there are a number of fully-trained nurses on the staff of these two hospitals, which is a very satisfactory condition of things, and we thank our correspondent for her pleasant letter.

### CHOLERA AND COMMON SENSE.

"Matron" says: The information given in last week's Hospital is especially interesting and valuable just now. I think your article, "From a Nurse's Point of View," is one of the best I have seen on the cholera question, and I cordially agree with the writer in advocating more common sense and less sensationalism in dealing with emergencies.

### CHOLERA PREPARATIONS AT THE HOSPITALS.

A letter from a "Metropolitan Hospital Nurse" says: I and many other nurses wish to thank Dr. Potter for his letter in the daily papers of Saturday. Feeling how well to the fore the hospitals have been in getting wards ready, and in organising their nursing staffs for any cholera emergencies, some of us felt sorry that no one had taken the trouble to let the Princess Christian know all this. She would not have thought there was any need to appeal to nurses through the newspapers if she had been told of the numbers who have already volunteered.

### HOSPITAL ARRANGEMENTS.

"Another Trained Nurse" writes: At our hospital we have a party of selected nurses ready for the cholera. We hope it won't come, of course, but when Matron asked those who wished to volunteer to send in their names, such a long list was rapidly made that she had to sort out those whom she thought most suitable. You see, sir, everybody likes to do something out of the common run of work, but our own sick folks and regular duties must be also attended to.

### AN OLD HAND.

"A Nurse Who Knows What Cholera Is" writes: I wish you would print a few words of warning to all those young women who are being unsettled about reports of cholera. Tell them to stick to their work and do their duty steadily. If cholera comes (I don't call these stray cases anything to frighten one), we know quite well that the doctors and the matrons have got everything settled, and there will be no bother at all. I am sure all this talk and fuss and sensation is far worse for nurses, especially young ones, than a little extra hard work would be. They are behaving like young soldiers who have never been under fire and are very brave beforehand, but become thoroughly demoralised when the guns go off.

### A PRIVATE NURSE'S REPORT.

From "An Institution Nurse" we hear: Of course, Dr. Potter is quite right in saying every preparation has been made in the hospitals, and I can add that this association, which is one of the fairest dealing ones in London, so far as justice to nurses goes, has some thirty fully-trained, steady nurses who could be relied upon at a moment's notice to go to any of the doctors, who always get their nurses there, and who would, of course, telegraph immediately if any case of necessity arose of cholera or any other equally urgent illness.

### GENERAL HOSPITAL, BIRMINGHAM.

We had not space to insert the letter of the "Late Sister Birmingham General Hospital" last week, though we referred to it. We are interested to hear her experience of the improvements which she had witnessed during Miss Busby's term of office there. We sincerely trust that the upward progress of the nursing department will be steadily continued, and that past and present workers will have every reason to be proud of their hospital.

### REMOVAL OF THE BRASSEY HOME.

"A Nurse from Liverpool" writes to regret the removal of the Brassey Home to the Isle of Wight. She says, truly, that the long journey precludes the possibility of nurses in the north availing themselves of its advantages. We share in her regrets that any place within a moderate journey from London and towns in the south of England should not be also accessible to our Liverpool sisters. There seems but little use in protesting against a step which has been already taken, but we hope our northern nurses will soon possess, in addition to their present advantages, such holiday homes, clubs, &c., as they may desire. We are always willing to give attention and publicity to any schemes set on foot for the real benefit of nurses.

### CHOLERA LECTURES TO NURSES.

"Margaret R. Nichol, Secretary to the Trained Nurses' Club, 12, Buckingham Street, W.C.," writes: In consequence of recent suggestions, we are arranging a series of classes for the instruction of trained nurses with regard to the nursing of cholera patients. I shall be glad to hear from nurses and others desiring to avail themselves of the above.

### Presentations.

On Saturday, August 20th, Dr. H. Elwin Harris, Medical Superintendent of St. Saviour's Infirmary, East Dulwich Grove, S.E., was presented with a handsome silver tea service from the whole staff of the Infirmary, with their congratulations and good wishes on his approaching marriage.

On August 1st, the decoration of the Royal Red Cross was bestowed upon Mrs. Watson (née Welchman), late of the Indian Nursing Service, for her distinguished assistance during the Hazara Expedition of 1888. Major-General Sir George White, K.C.B., K.C.I.E., V.C., Commanding at Quetta, under special instructions from the Queen-Empress, made the presentation with much ceremony before a brilliant assemblage of civil and military society. Sir George White remarked, in the course of his very pleasant speech, that this was the second "soldier's wife" who had gained this coveted distinction. Lady Roberts being the first.

### Death in our Ranks.

We announce with sincere regret the death of Nurse Elizabeth Hook, aged 26 years, at the Royal Infirmary, Preston, from scarlet fever, contracted in the discharge of her duties in the infectious wards.

### Appointments.

[It is requested that successful candidates will send a copy of their applications and testimonials, with date of election, to The Editor, The Lodge, Porchester Square, W.]

Government Hospital, Gibraltar.—On August 12th Miss Louise Maud Mantle sailed for Gibraltar, where she has been appointed Sister at the Government Hospital. Miss Mantle was trained at the London Hospital, and we wish her all success in her new sphere of work.

Mater Misericordiæ Hospital, Dublin.—Miss Mary McGivney has been appointed Matron of the new Nursing School established at this hospital. Miss McGivney was trained at the London Hospital, where she proved herself an excellent and conscientious nurse, and we have great pleasure in congratulating her on her promotion to so important a post.

Market Drayton Cottage Hospital.—Miss Kate E. Richards has been appointed to this hospital, which was opened last July. She was for some years a Sister at the Birmingham General Hospital, where she earned excellent testimonials. She afterwards held the post of Matron at Berkeley Cottage Hospital for nearly a year and a-half, giving universal satisfaction, and we beg to congratulate her on her present appointment, and also the committee on having secured a popular and experienced nurse for the new hospital.

St. Mary's Hospital for Women and Children, Manchester.—Miss Clara Tibbits, who has just been appointed Matron of this hospital, was trained at the Royal Infirmary, Manchester, where she afterwards held the post of Night Superintendent for one year. Miss Tibbits was then made "Home Sister," and occupied that position for two years. We cordially congratulate her on her present appointment, for which her training has specially qualified her.

### Hints to Nurses.

### FOLDING BED.

The folding bed which we have seen at 28, Hart Street, Bloomsbury, is especially adapted for private nurses who are able to furnish a room, and thus keep for themselves a miniature home between their cases. When not in use this "Standard" bed is converted into the semblance of a cabinet or portière, and in both cases it is put away quite ready, so that the removal of the straps which have kept the bedding in place is the only thing needful after the spring mattress has been put into position. It is a simple, cleanly, and hygienic arrangement, for air is freely admitted, and there are none of the drawbacks of closeness and stuffiness which are generally associated with "shut-up" bedsteads. When out of use it forms a pretty piece of furniture, very suitable for a hospital sister's single room; and when required, it forms a comfortable and reliable resting-place—a complete and comfortable bed, not a ricketty make-shift couch.

### Notes and Queries.

### Queries.

*Paralytic.*—Can any of our readers suggest a home for a patient aged 67, where he could be kindly looked after? He can only pay £20 per annum. He says he is not quite helpless, and his intellect is unimpaired.

Would you, or one of your correspondents, kindly inform me whether the Local Government Board hold a trained staff, consisting of superintendent and nurses as first and second class officers; and also if it is possible to obtain the rules drawn up by the Local Board for their guidance?—*Fair Play.*

### Answers.

*Kattileen.*—You are too young for any general hospital, but some infirmaries and several children's hospitals take probationers of twenty-one. Do you wish to be trained in London, or would you be willing to go to a country town? If you write to any matron, she will send you the rules regarding admission of probationers. Twenty-five is the regular age in large hospitals. Pray write again if you want any more information.

*Alice.*—Write to Hon. Sec., Glasgow Nurses' Co-operation, for the information required. She will either give it or tell you to whom to apply.

*A. B.*—Read Dr. Harding's lectures, now coming out in "Nursing Mirror," and any books on the nursing of mental cases.

*Scotia.*—Please send name and address if you want your questions answered.

*W. A. H.*—Your questions shall be answered in a day or two.

### The Result of a Chill.

### I.—THE HERMITS OF DARTMOOR.

One league south of a lone morass, the birthplace of many noble Devon rivers, is an old homestead known for many generations as Hollacombe. It is in one of the deep valleys cleft by the turbulent currents that merge into the Teign, sheltered east and west from moorland tempests by barren mountain ridges, and built of rough, honest Dartmoor granite, warmed with age to pink and bronze. Brown ivy clothes the wide porch, the moss on the thatch is the colour of an old green velvet hunting coat, and the smoke from the tall chimney piles is blue against sombre slopes. Time was when the glebe on the southern down yielded stunted turnips for the yeoman's vigorous combat with deep-rooted gorse and hordes of coneys, but Nature has regained dominion over the rocky soil, and golden brakes and tangles of whortleberry hide all traces of former tillage. Still, ponies, mutton, and game have paid the rent of Hollacombe, though crops have failed, for there are many miles of grazing land within the stone fence that bounds the estate, and big mixed bags have been secured by the lessee of the shooting rights.

Three years ago the lord of the manor, Sir Nicholas Purchase, an Alderman of London, received an application for the tenancy of Hollacombe. The barton had been vacant for two years, and Sir Nicholas was willing to let it, without the land, at a much reduced rental. From John Newcombe, his agent, he learnt that the would-be tenants were two young single ladies, sisters, of evident respectability, who desired to settle themselves in that district in order to paint views of the scenery. Accompanying his note were references from a City tea broker and a retired Inspector-General of Naval Hospitals, stating that the Misses Ruthven, being of good connections, might be in every way regarded as trustworthy tenants. Upon reading this the Baronet directed that the house should be put into repair at once, suggesting that Newcombe should make all alterations deemed necessary to the comfort of the ladies.

Six weeks after, the new tenants of Hollacombe were seen shopping in Chagford. In this thinly-peopled district tongues are set going glibly at the advent of strangers. The grocer told the baker's wife that the young ladies up at Hollacombe were pleasant spoken, somewhat queerish in their dress, and apparently reduced in circumstances. The last piece of information was speedily corroborated on all sides when it was discovered that the Misses Ruthven kept no kind of carriage—not even a pony-trap. This did not, however, affect their credit, because it was soon ascertained that all accounts were met with promptness at each week's end; nor was the fact that the strangers were decidedly ladylike disputed by any of the townsfolk. Still, they were accounted odd; and to live in any abnormal fashion, where people are sparse, inquisitive, and eminently normal, frequently induces conjecture, suspicion, and fabricated explanations of the mystery with which the rustic imagination loves to endue a new resident. It was passing strange that two attractive young ladies should immure themselves in a lonely farmhouse on the borders of Dartmoor, and seldom see a soul but the boy who came daily from the nearest cottage to chop their firewood. Ostensibly, they were "painting ladies," but that sort of feminine eccentric usually lived in apartments for a few weeks in summer, and then went back to London with a stack of pictures. But after a while the wonder subsided, and the Misses Ruthven came and went without so much as a window-blind being pulled aside to peer at their practical home-made wear of tweed.

Yet, though the girls ceased to cause curiosity among their scattered neighbours, they were interesting problems to themselves. They had come down to Devonshire from a dank, genteel square in Kennington, to study natural scenery and perfect their "composition." The elder sister, Sybil, now in her twenty-fifth year, was indubitably the more zealous of the pair. Though Hilda was not wanting in a certain degree of enthusiasm and energy, her sister was a positive fanatic of art. Having learned the rudiments of painting in a school of art, where they had both won prizes, and upon a preposterous marriage of Mrs. Ruthven to an ignoble and adipose distiller of Hackney, the girls had resolved to leave home and support themselves by painting. Sybil was the possessor of thirty pounds, the bequest of a relative; so with this, and a small collection of Chinese mats, vases, and images purchased by their father, the late Captain Ruthven, on his voyages, the girls entered upon their hazardous and Bohemian career.

The first six months at Hollacombe was a time of novel delights to the enthusiasts. Fine subjects for their brushes were to be found within a few paces from the door; the strong air gave their faces a bright, ruddy tint, their appetite for their plain fare increased amazingly, and their minds had healthful expansion in the daily contemplation of untamed nature. Dartmoor, with its grand stony ranges, sweeps of heathy down, and cleaves musical with the song of rippling streams, was a land of ravishing entrancement, after Kennington, with its straight, grimy streets, and discord of traffic.

It was sweet to rise soon after the sun on a spring morning, and see the moor glow with a wealth of warm hues, the hill-tops touched with gold, and the valleys vague in the purple haze of dawn. Day by day the savage charm of the scenery grew upon them, and imbued their minds with a kind of reverential adoration. Their life seemed like the even motion of a boat upon a silent stream; there were no distractions, no forebodings of trouble. It was as though fate had assured them perfect and permanent happiness, and complete health for such time as they willed to stay in this elysian region.

Yet, despite scrupulous eking of the thirty pounds of working capital, the sum began to sink to a ridiculous residuum, and to cause occasional qualms for the future. The first quarter's rent of a few pounds was cheerfully and promptly paid, but the second settlement seemed a more serious disbursement. Winter was approaching, expenses would be greater, and, besides this, they had heard somewhat discomfiting tales of Hollacombe being inaccessible to tradesmen's carts during snow. Before leaving London they had obtained a picture-dealer's promise to inspect their landscapes with a view to purchase. He could not, he said, entertain the idea of buying the work of unrecognized artists offhand; but if the pictures suited his patrons, he would, of course, be pleased to negotiate.

(*To be continued.*)

### Wants and Workers.

The Lady Superintendent of the Glasgow Sick Poor Nursing Association, will be grateful for parcels of old household linen and calico under-garments of all kinds, for which there is always urgent need. They should be addressed *Lady Superintendent*, 218, *Bath Street, Glasgow.*

Wanted, immediately, in the neighbourhood of Manchester or Leeds, a permanent home with Churchpeople, for a lady, many years powerless from spinal injury. An airy bed-room, sitting-room, or two rooms on ground floor, to avoid carrying. Also accommodation for spinal carriage. Terms must be moderate. Address *Sister Lucie, Littleborough, Lancashire.*

## Figures and Tables

**Figure f1:**